# TRAPPopathies: Severe Multisystem Disorders Caused by Variants in Genes of the Transport Protein Particle (TRAPP) Complexes

**DOI:** 10.3390/ijms252413329

**Published:** 2024-12-12

**Authors:** Riley Hall, Vallari Sawant, Jinchao Gu, Tim Sikora, Ben Rollo, Silvia Velasco, Jinkuk Kim, Nava Segev, John Christodoulou, Nicole J. Van Bergen

**Affiliations:** 1 Murdoch Children’s Research Institute, Melbourne, VIC 3052, Australia; riley.hall@mcri.edu.au (R.H.); vallari.sawant@mcri.edu.au (V.S.); tim.sikora@mcri.edu.au (T.S.); silvia.velasco@mcri.edu.au (S.V.); john.christodoulou@mcri.edu.au (J.C.); 2 Department of Paediatrics, University of Melbourne, Melbourne, VIC 3052, Australia; 3 The Novo Nordisk Foundation Center for Stem Cell Medicine, reNEW, Murdoch Children’s Research Institute, Melbourne, VIC 3052, Australia; 4 Department of Neuroscience, School of Translational Medicine, Monash University, Melbourne, VIC 3000, Australia; jinchao.gu@monash.edu (J.G.); ben.rollo@monash.edu (B.R.); 5 Graduate School of Medical Science and Engineering, Korea Advanced Institute of Science & Technology (KAIST), Daejeon 34141, Republic of Korea; jinkuk@kaist.ac.kr; 6 Department of Biochemistry and Molecular Genetics, University of Illinois, Chicago, IL 61801, USA; nava@uic.edu

**Keywords:** TRAPP complex, neurological disease, Rab GTPase, human genetics, pediatrics, intracellular trafficking, autophagy

## Abstract

The TRAPP (TRAnsport Protein Particle) protein complex is a multi-subunit complex involved in vesicular transport between intracellular compartments. The TRAPP complex plays an important role in endoplasmic reticulum-to-Golgi and Golgi-to-plasma membrane transport, as well as autophagy. TRAPP complexes comprise a core complex, TRAPPI, and the association of peripheral protein subunits to make two complexes, known as TRAPPII and TRAPPIII, which act as Guanine Nucleotide Exchange Factors (GEFs) of Rab11 and Rab1, respectively. Rab1 and Rab11 are GTPases that mediate cargo selection, packaging, and delivery during pre- and post-Golgi transport in the secretory pathway. Rab1 is also required for the first step of macroautophagy, a cellular recycling pathway. Pathogenic variants in genes encoding protein subunits of the TRAPP complex are associated with a range of rare but severe neurological, skeletal, and muscular disorders, collectively called TRAPPopathies. Disease-causing variants have been identified in multiple subunits of the TRAPP complex; however, little is known about the underlying disease mechanisms. In this review, we will provide an overview of the current knowledge surrounding disease-associated variants of the TRAPP complex subunits, propose new insights into the underlying disease pathology, and suggest future research directions into the underlying disease mechanisms.

## 1. Introduction

Precisely controlled movement of cargo within cells is necessary for eukaryotic life. Disruption to pathways that regulate the formation, transport, and fusion of vesicles compromises proper cell function. Ypt/Rab GTPases are a protein family of molecular switches that mediate these important trafficking pathways. They are inactive in the cytosol, but when they reach their target membrane, Ypt/Rab GTPases are activated by a family of proteins known as Guanine Nucleotide Exchange Factors (GEFs), which promote attachment to the cell membrane [1]. Thus, the role of GEFs is to control the spatial–temporal activation of Ypt/Rabs, which is achieved by catalyzing the exchange of bound GDP for GTP. This conversion permits the interaction of Rab GTPase with effector proteins that are involved in cargo selection, as well as vesicle formation and delivery. These interactions are vital for the correct packaging, transport, and delivery of proteins and lipids to the cell membrane or their target location within the cell [1,2].

More than 60 Rab GTPases have been identified in the human genome, and while multiple GEF families have been identified, the complementary GEF for many Rabs are still unknown. This may be due to the low homology between known GEF family members.

The TRAPP (TRAnsport Protein Particle) family of proteins forms two separate GEF complexes, TRAPPII and TRAPPIII, which activate Rab11 and Rab1, respectively [3]. Rab1 plays a key role in the regulation of endoplasmic reticulum (ER)-to-Golgi transport and the formation of autophagosomes from the ER, while Rab11 regulates post-Golgi secretion and endosomal recycling [4]. The two TRAPP complexes share an identical TRAPP core, but each subcomplex has a unique set of accessory proteins that confer specificity for a unique Rab GTPase.

The first TRAPP subunit recognized, TRAPPC3, was identified in yeast (homolog; Bet3) during a screening of components of the secretory pathway [5], and the first TRAPP protein complex was identified soon after [6]. Since then, a multitude of biochemical and cellular studies have been conducted to unravel the structural and functional properties of TRAPP subunits and complexes. More recently, advances in next-generation sequencing (NGS) technologies have enabled the identification of disease-causing TRAPP variants in almost all of the TRAPP subunits. However, there are still large gaps in our understanding of how these genetic variants manifest as the severe disorders seen in the clinic.

Disorders caused by variants in the TRAPP subunits are collectively known as TRAPPopathies [7]. Affected individuals present with common but also discrete phenotypes, even though all subunits are involved in the same intracellular pathways. TRAPPopathies are generally characterized by severe neurological and/or musculoskeletal symptoms, which typically arise early in life. While many TRAPPopathies are severe monogenic disorders, multiple TRAPP variants have been linked to polygenic and progressive diseases, such as various cancers and Alzheimer’s disease, as outlined below.

Given the high conservation of TRAPP proteins across all eukaryotes, yeast cells have been used as a model to study structural and functional aspects of TRAPP complexes. However, to understand the pathophysiology of human TRAPPopathies, more complex models are required that recapitulate pathogenic variants in human cells or tissues. Despite improvements in identifying and diagnosing the TRAPPopathy spectrum of disorders with NGS technologies, little is known about their underlying pathophysiology. It is also not understood why some TRAPP complex subunits are essential for life, whilst others appear to be dispensable. Lastly, there are currently no effective therapeutics available for TRAPPopathy patients, which may be explained in part by a poor understanding of underlying disease mechanisms and the lack of appropriate targets.

Previous reviews on TRAPP complexes have outlined the structure and biochemical role of the TRAPP subunits [4,8], and the most recent review investigating TRAPPopathies specifically was published in 2019 [7]. The previous review provided a comprehensive overview of the genotype–phenotype correlations of the TRAPP complex, coined the term “TRAPPopathy” to collectively describe TRAPP-related disease, and provided novel insights into the function of the TRAPP complex. Since 2019, there have been multiple new disease associations with the TRAPP complexes, including TRAPPC1, TRAPPC4, TRAPPC10, and TRAPPC14, expanding our previous understanding of disease mechanisms and molecular functions of the TRAPP complexes. This review focuses on the genetic and pathophysiological spectrum of human TRAPPopathies, summarizing the disease models that have been used to understand the function of each subunit and highlighting areas that require further exploration. We also propose methods that may fast-track the development of new treatments to improve clinical care for affected individuals.

## 2. The TRAPP Complexes

### 2.1. Core TRAPP

The protein subunits of the TRAPP core include TRAPPC1, TRAPPC2, TRAPPC2L, TRAPPC3, TRAPPC4, TRAPPC5, and TRAPPC6 [4]. The yeast homologs for these subunits are Bet5, Trs20, Tca17, Bet3, Trs23, Trs31, and Trs33, respectively (Table 1). In humans, these subunits form a TRAPP octamer core, with two TRAPPC3 subunits present. TRAPPC1 and TRAPPC4 form the catalytic site of the core and are located in the center of the rod-like structure of the complex.

Although TRAPPII and TRAPPIII activate unique Rab GTPases, the TRAPP core is the site of activation for both complexes. Thus, the specificity of activation might be mediated through accessory subunits. In vitro, the core complex is sufficient to activate Rab1 alone [9]. However, in vivo activation of Rab1 and Rab11 requires the accessory proteins of TRAPPII and TRAPPIII.

### 2.2. TRAPPII

**Figure 1:**
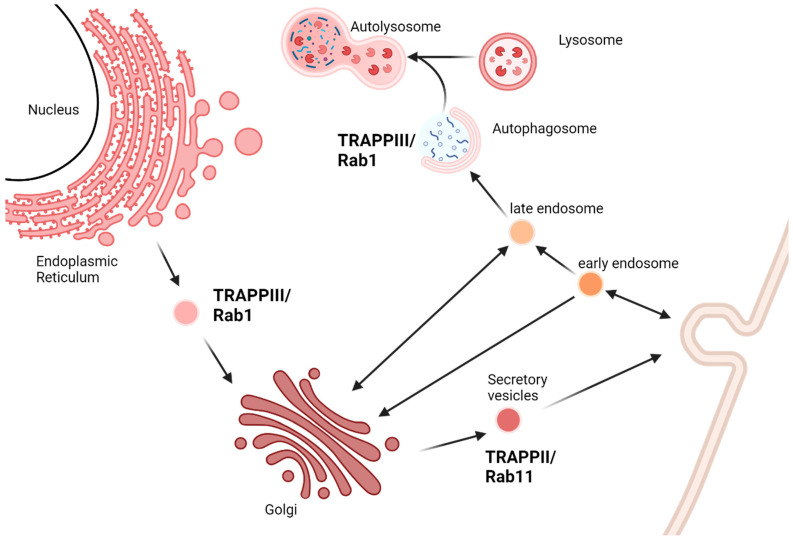
Stages of the vesicular transport pathway in which TRAPPII and TRAPPIII and their respective Rab GTPases are involved. The TRAPPIII complex is a Guanine Exchange Factor (GEF) for Rab1, which mediates early stages of the vesicular transport pathway from the endoplasmic reticulum (ER) to the Golgi apparatus and formation of autophagosome membranes prior to fusion with lysosomes into the autolysosome. The TRAPPII complex is a GEF for Rab11 and mediates the Golgi-to-plasma membrane stage of the secretory pathway. Created in BioRender [18].

The TRAPPII complex includes the TRAPP core, as well as accessory proteins TRAPPC9 and TRAPPC10 [4,10]. Yeast TRAPPII includes the TRAPP core and the TRAPPII-specific subunits Trs120 (TRAPPC9 homologue) and Trs130 (TRAPPC10 homologue), as well as a unique yeast subunit, Trs65, with no known human homologue [11]. TRAPPII activates Rab11 in humans and the yeast homologues Ypt31 [12] and Ypt32 [13,14], which are involved in steps of the post-Golgi secretory pathway, endosomal recycling, and autophagy (Figure 1). TRAPPII and Rab11 are involved in ciliogenesis [15], a critical pathway in neuronal development [16]. In addition to TRAPPII, Rab11 can be activated by another unrelated GEF in Metazoans, and they appear to have unique cellular functions [17]. This redundancy means that TRAPPII-specific subunits are essential for cell viability in yeast, but not in Metazoans [9].

### 2.3. TRAPPIII

In addition to the TRAPP core, the Metazoan TRAPPIII complex also includes accessory proteins TRAPPC8, TRAPPC11, TRAPPC12, and TRAPPC13 [4]. In yeast, there is only one TRAPPIII accessory subunit, Trs85, the homolog of TRAPPC8. TRAPPIII activates Rab1 and is the only identified GEF for this Rab GTPase [19], which is a regulator of ER to Golgi transport. TRAPPIII and Rab1 also have a critical role in cell autophagy (Figure 1). Specifically, TRAPPIII is involved in COPII vesicle tethering to the pre-autophagosomal structure [20,21].

## 3. TRAPP Subunits and Human Disease-Causing Variants

### 3.1. Core TRAPP Subunits

Core TRAPP subunits are common to both TRAPP complexes that activate both Rab1 and Rab11. Therefore, pathogenic variants in TRAPP subunits of the core may have overlapping pathologies.

#### 3.1.1. TRAPPC1

TRAPPC1 is a member of the TRAPP core and is involved in the activation of both Rab1 and Rab11, facilitating both early and late stages of the Golgi transport pathway. TRAPPC1 is ubiquitously expressed in all human tissues. The yeast homologue is known as Bet5 or MUM2, and both are essential for yeast and human cell viability.

Depletion of TRAPPC1 has been linked to effects on the immune system. Specifically, in 2022, Dong et al. explored the vital role of TRAPPC1 in Thymal Epithelial Cell (TEC) development and maturation [22]. A TEC-specific *Trappc1*-deletion mouse model was generated by crossing *Trappc1*-floxed mice with *Foxn1*-cre mice. This study generated both *Trappc1* deletion and *Trappc1* heterozygous mice, although a high mortality rate was observed in the *Trappc1* deletion mice. Consequently, investigations were conducted in neonatal mice. *Trappc1* deletion mice showed poor thymic T cell output and a higher rate of spontaneous autoimmune disorders [22]. Further evidence that TRAPPC1 has a role in T-cell development and homeostasis was demonstrated [23]; *TRAPPC1* knockout naïve T cells showed an increase in ferroptosis-related, damage-associated molecular pattern molecules.

Recently, biallelic *TRAPPC1* variants were linked to a severe neurodevelopmental disorder and myopathy in one patient [24]. These variants were studied in a humanized yeast model. One variant was demonstrated to be non-functional whilst the other variant was conditional-lethal in a temperature-dependent manner and impaired autophagy and secretion above 35 °C, potentially explaining its human pathogenicity. Similar impairments were identified in patient fibroblasts, demonstrating the utility of the humanized yeast model in studying human variants of uncertain significance in TRAPP-related disorders in situations where primary patient cells may not be available [24]. Two *TRAPPC1* variants of uncertain significance are listed in ClinVar [25].

#### 3.1.2. TRAPPC2

TRAPPC2 is ubiquitously expressed across all human tissues, is part of the TRAPP core, and is required for the correct assembly of both TRAPPII and TRAPPIII [26], likely acting as an adaptor for TRAPPC8 and TRAPPC9 in the TRAPPII complex [27]. Yeast studies identified that TRAPPC2 connects the TRAPP complexes to a subunit necessary for exit of oversized cargo, including procollagen, from the ER [26], because it forms prefibrils that are too large to fit into typical vesicles. Instead, a specific interaction of TRAPPC2 with TANGO facilitates the export of procollagen. Thus, pathogenic variants in *TRAPPC2* may underly the skeletal disorder Spondyloepiphyseal Dysplasia Tarda (SEDT) due to defective chondrogenesis [28].

##### Skeletal Disorder

SEDT is a human heterogeneous progressive X-linked skeletal disorder characterized by a barrel chest; dysplasia of large joints; and a short neck, trunk, and stature [29,30,31]. SEDT is caused by a range of pathogenic variants in the *TRAPPC2* gene (previously named *Sedlin* or *SEDL*), and was first identified as an X-linked disorder in 1999 [31]. The sequence of the *Sedlin* gene and its role in ER-to-Golgi transport were identified in the same year [32], shortly after the TRAPP complexes were first identified [6]. TRAPPC2 was identified as the ortholog of the yeast Trs20 protein [33]. Whilst there is a comprehensive list of known *TRAPPC2* disease-causing variants associated with SEDT [34], there are no effective therapeutics available for patients with care limited to various palliative surgeries [35]. There is a recommendation that SEDT patients avoid activities which may increase damage to their bones.

*TRAPPC2* is located on the X chromosome, and it has been determined that the *TRAPPC2* gene escapes X-inactivation [36]. This suggests that haploinsufficiency is not enough to cause an SEDT phenotype in female carriers. Most pathogenic variants in *TRAPPC2* severely truncate the protein or eliminate the protein entirely.

While diagnosis of SEDT has been made possible by recent advancements in genetic technologies, there are still major knowledge gaps in our understanding of this complex heterogeneous disease. While clinical reports of SEDT are abundant, pre-clinical studies and current disease models have proven insufficient in advancing our understanding of the pathophysiological processes and potential treatment strategies for this disease.

Since TRAPPC2 is a core TRAPP complex protein, it is likely that TRAPPC2 has a specific independent role, which may explain its skeletal-specific phenotype. In this respect, TRAPPC2 binds and regulates cycling of Sar1, a GTPase involved in membrane constriction around procollagen fibrils, in collaboration with TANGO1, promoting the export of procollagen from the ER [28]. An impairment in procollagen secretion could lead to accumulation of unfolded collagen proteins in the ER, triggering the unfolded protein response. To date, no other TRAPP gene has been associated with a skeletal disorder, and no other phenotypes have been associated with *TRAPPC2* variants.

#### 3.1.3. TRAPPC2L

TRAPPC2L is a subunit of the TRAPP core, but only shares ~22% sequence homology with TRAPPC2. It is required for the binding of the TRAPPII- and TRAPPIII-specific subunits to the core [37].

##### Neurodevelopmental Disease

Two homozygous missense variants [38,39] and one homozygous nonsense variant [40] have been linked to TRAPPC2L-related neurodevelopmental disorders. Patients with *TRAPPC2L* pathogenic variants exhibited febrile illness-induced encephalopathy, neurodevelopmental delay, rhabdomyolysis, epilepsy, and tetraplegia. Milev et al. used whole-exome sequencing (WES) to identify a homozygous pathogenic variant in *TRAPPC2L* in two unrelated patients with similar neurodevelopmental phenotypes and a NM_001318525.2(*TRAPPC2L*):c.109G>T;p.(Asp37Tyr) variant [39]. Biochemical and functional genomics in patient fibroblasts and yeast confirmed that the missense variant in *TRAPPC2L* interfered with the binding of the TRAPP core to the TRAPPII-specific subunit, TRAPPC10. Patient fibroblasts with the p.(Asp37Tyr) protein variant had an intracellular trafficking defect. Haplotype analysis suggested a founder effect for this variant. This variant also increased the levels of activated Rab11 and reduced cilia formation.

A second missense variant NM_001318525.2(*TRAPPC2L*):c.5C>G;p.(Ala2Gly) was identified in three unrelated siblings with global developmental delays [38]. Membrane trafficking assays confirmed that transport to and from Golgi was delayed, and size exclusion chromatography suggested that the variant prevented correct formation of the TRAPP complexes. A yeast two-hybrid assay and in vitro binding indicated that this variant specifically affects the interaction between TRAPPC2L and the TRAPP core subunit TRAPPC6a.

More recently, the pathogenic variant spectrum of *TRAPPC2L* has been expanded, where the first protein truncating pathogenic variant NM_016209.3(*TRAPPC2L*):c.367C>T, NP_057293.1;p.(Gln123Ter) was identified in two siblings with hypotonia, developmental delay within the first few months of life, mild facial dysmorphism, joint hypermobility, and non-specific brain MRI abnormalities [40]. Elevated levels of creatine kinase were seen in both patients, suggesting that this variant also involves neuromuscular degradation. Examination of ClinVar revealed a range of different variant types listed as pathogenic, mostly missense, but also some intronic and frameshift. These variants were associated with progressive, early-onset encephalopathy with episodic rhabdomyolysis. Overall, the majority of *TRAPPC2L* variants appear to be loss-of-function variants with autosomal recessive inheritance.

Whilst the clinical presentation of TRAPPC2L-related disorders is well-characterized, the biological processes of these symptoms are not understood. Cellular studies have been limited to gene-validation studies in patient fibroblast cells and yeast models, which do not recapitulate the neuromuscular and neurodevelopmental phenotype associated with this disease.

#### 3.1.4. TRAPPC3

TRAPPC3 is a member of the TRAPP core complex and is essential for stabilizing the TRAPP complex and facilitating vesicle tethering and transport. TRAPPC3, -C9, and -C10 physically interact and function with Bardet–Biedl syndrome (BBS) proteins to mediate primary cilia biogenesis in retinal pigment epithelial cells [15]. Recently, genomic approaches were applied to a large cohort of patients with a ciliopathy phenotype, and a homozygous missense variant in *TRAPPC3* was identified as a novel ciliopathy candidate gene, but was only identified in one ciliopathy patient [41]. ClinVar lists one missense variant, NM_014408.5(*TRAPPC3*):c.184C>T;p.(Arg62Trp), as likely pathogenic, associated with BBS, and this patient was reported to have obesity, developmental delay, poor vision and polydactyly.

To date, there are no other reports of pathogenic variants in *TRAPPC3*, and no disease models have been specifically generated for TRAPPC3.

#### 3.1.5. TRAPPC4

TRAPPC4 is a component of the TRAPP core and forms part of the catalytic site for both TRAPPII and TRAPPIII [4]. The yeast homolog is Trs23 (Synbindin). TRAPPC4 was first identified in 2000, colocalizing with the cell surface heparan sulfate proteoglycan, syndecan-2, in hippocampal dendritic spines [42]. The interaction between TRAPPC4 and syndecan-2 was confirmed by yeast two-hybrid studies and co-immunoprecipitations. To understand the physiological significance of this interaction in a neuronal context, rat hippocampal neurons were transfected with TRAPPC4-GFP alone or in combination with syndecan-2. When TRAPPC4-GFP was transfected alone, the protein was distributed diffusely across the cytoplasm, but upon co-transfection with syndecan-2, TRAPPC4 localized to dendrites [42]. As syndecan-2 has previously been implicated in the maturation of dendritic spines [43], this suggests that TRAPPC4 plays a role in dendrite maturation by recruitment of the correct intracellular vesicles. Importantly, RT-PCR and in situ hybridization experiments have demonstrated high expression of TRAPPC4 in large pyramidal neurons, providing further support for the requirement for TRAPPC4 in dendrite maturation in these cell types [42].

##### TRAPPC4 in Neurodevelopmental Disorders

More recently, TRAPPC4 was linked to a rare neurodevelopmental disorder, where an autosomal recessive pathogenic variant was identified in three unrelated families via WES and cell-based functional analyses [44]. Children presented with a severe neurodevelopmental syndrome, progressive cerebellar and cortical atrophy, early-onset seizures, spastic quadriparesis, microcephaly, and severe intellectual disability early in life. The homozygous *TRAPPC4* variant identified NM_016146.6(*TRAPPC4*):c.454+3A>G to be located at a well-conserved splice site in the *TRAPPC4* gene. This variant results in a leaky splicing effect, where the *TRAPPC4* transcript is mis-spliced, with reduced expression of wild-type transcript. The low wild-type transcript levels translate to low levels of TRAPPC4 protein and partial TRAPP complex assembly defects [44]. More in-depth RNAseq analysis of a patient fibroblast line revealed that exon 3 of the TRAPPC4 transcript was skipped in 46% of transcripts [45]. Interestingly, the exon-skipping event was also present in 3% of reads in control fibroblasts. Additionally, there was generation of an aberrant transcript owing to the presence of a downstream cryptic splice donor site [45]. It is known that TRAPPC4 is essential for life, as complete knockout of the gene is embryonic-lethal in mice [46]; however, the reduced level of wild-type TRAPPC4 in patients with the c.454+3A>G variant is sufficient to sustain life. Since the TRAPP complexes are involved in intracellular trafficking pathways, secretory pathways were examined using a temperature-sensitive form of Vesicular stomatitis virus G (VSV G) fused to GFP (VSV-G-ts045-GFP) as a marker for protein trafficking through the Golgi. A delay in trafficking to and from the Golgi was found in patient fibroblasts compared to control fibroblasts. Defects in basal and starvation-induced autophagic flux were also demonstrated in patient fibroblasts [44].

Approximately 25 more cases with the same c.454+3A>G variant have been identified. Most patients present with a similar phenotype as previously reported, exhibiting severe psychomotor delay, microcephaly, progressive spastic tetraplegia, early-onset epilepsy, and developmental regression [45,47,48]. A handful of patients have been reported with elevated creatine kinase and blood lactate, with muscle involvement in addition to the neurological phenotype [47,49]. Most affected individuals showed a partial response to anti-seizure medication, and one patient had osteopenia and premature adrenarche, signs of hypothalamic dysregulation [45].

The novel homozygous missense variants (NM_016146.6(*TRAPPC4*):c.191T>C;p.(Leu64Pro) and NM_016146.6(*TRAPPC4*):c.278C>T;p.(Pro93Leu)) have been reported in three individuals from two Indian families [49]. The patient who was homozygous for the c.191T>C variant presented with an early infantile neurodevelopmental phenotype consistent with previous reports of TRAPPC4-associated disorders. However, two siblings homozygous for the c.278C>T variant presented with cognitive delay in early childhood, late-onset regression of achieved milestones, and microcephaly. In silico analyses of the p.(Leu64Pro) variant predicted destabilization of the core structure of the protein and decreased protein stability, whilst the p.(Pro93Leu) variant was predicted to alter a residue on the surface of TRAPPC4 that may destabilize internal protein interactions, altering spatial conformation of the protein.

In summary, TRAPPC4 is classified as an autosomal recessive disease, and complete loss of function is incompatible with life. We know that the founder pathogenic variant c.454+3A>G responsible for the majority of cases is a hypomorphic (leaky) pathogenic variant that retains some protein functionality, even in homozygotes, and the other reported missense variants above retain some functionality. Additionally, there are other reported pathogenic variants for this gene listed in ClinVar with autosomal recessive inheritance. For example, p.(Leu125Pro) and p.(Gly213Ter220delinsXaa) are reported for an autosomal recessive neurodevelopmental disorder with epilepsy, spasticity, and brain atrophy; the latter variant has a somewhat milder phenotype than that of patients with the c.454+3A>G variant. Therefore, it is likely that more pathogenic variants in *TRAPPC4* may be identified.

Despite the recent evidence that TRAPPC4 deficiency is associated with a neurodevelopmental disorder, disease modelling advancements to further elucidate the neurological phenotype have not yet been attempted, with current functional testing being limited to patient fibroblasts and yeast models.

##### TRAPPC4 in Cancer

**Figure 2:**
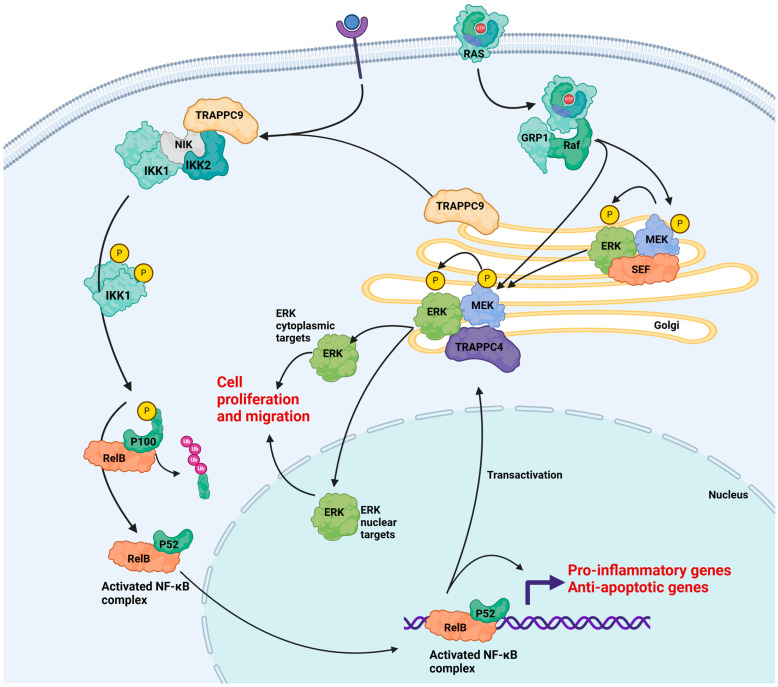
The role of TRAPPC4 and TRAPPC9 in cell signaling. TRAPPC4 (Synbindin) is involved in the Ras-Raf-Mek-ERK signaling pathway. RAS translocates from the plasma membrane to the Golgi where association with GRP1 promotes a phosphorylation cascade of MEK and ERK. TRAPPC4 binds to MEK1 and ERK in the Golgi after phosphorylation of ERK2 by MEK and activation of nuclear and cytoplasmic ERK targets [52]. ERK targets cause enhanced cell proliferation and migration in cancer [55]. TRAPPC9 functions within the NFκB pathway by binding to NIK and IKK2, leading to phosphorylation of IKK1, which then phosphorylates P100, promoting P100 cleavage and processing of P100 to P52, which, in conjunction with RelB, forms the activated NFκB complex [52,55,56,57]. Figure adapted from [52,58] and created in BioRender [59].

Beyond the role of TRAPPC4 deficiency in neurodevelopmental disorders, fundamental research in the cancer field has uncovered links between TRAPPC4 dysregulation and carcinogenesis. Multiple reports have revealed that TRAPPC4 interacts with vital proteins in cancer-causing pathways and contributes to the molecular changes seen in cancer cells. TRAPPC4 has been identified as an Extracellular Signal-regulated Kinase 2 (ERK2) binding factor [50,51,52]; has been shown to associate with the transcription factor NF-kB, which regulates both the innate and adaptive immune responses (Figure 2); and is a regulator of the Programmed Death Ligand-1 (PD-L1) [53]. TRAPPC4 has been shown to be differentially regulated in colorectal cancer [46,51,52,53,54].

#### 3.1.6. TRAPPC5

TRAPPC5 is another core protein of the TRAPP complex and is essential in yeast and in humans. It has a close interaction with TRAPPC3 and stabilizes the binding of TRAPPC8 to the core. TRAPPC5 also binds Rab proteins by their hypervariable domain, which accelerates the GTP to GDP process significantly.

There are no reported pathogenic variants in *TRAPPC5* in ClinVar. One report identified upregulation of *TRAPPC5* in hepatocellular carcinoma (HCC) subsequent to knockout of *CCT4*, a key component of HCC glycolytic metabolism [60]. However, there are no reported disease associations with TRAPPC5.

#### 3.1.7. TRAPPC6A/B

TRAPPC6 is part of the TRAPP complex core. In humans, there are two coding genes for *TRAPPC6*—*TRAPPC6A1* and *6A2*—that arise from one gene and vary by a 14-amino acid stretch, and *TRAPPC6B* from a second gene. There is ~55% homology between *TRAPPC6A* and *TRAPPC6B* in humans. The yeast homologue to *TRAPPC6*, *Trs33*, is part of the core TRAPP complex.

##### Neurodevelopmental Phenotype

A recessive variant, NM_001270891.2(*TRAPPC6A*):c.277T>A;p.(Tyr93Asn), was identified in five individuals of a consanguineous family of Saudi origin with intellectual disability, speech delay, facial dysmorphism, and polydactyly [61]. The variant is still classified as a Variant of Uncertain Significance (VUS). The variant was predicted to alter protein stability. This was tested by overexpression studies in HEK293T cells, where negligible levels of wild-type TRAPPC6A protein were detected compared to the p.(Tyr93Asn) variant. Treatment with the proteosome specific inhibitor MG132 resulted in an increase in the wild-type protein, suggesting that the wild-type protein is usually targeted for ubiquitination and degradation. The accumulation of the mutant protein was predicted to adversely affect protein trafficking, but this has not been functionally validated. There are no pathogenic variants for *TRAPPC6A* reported in ClinVar.

The first pathogenic variant in *TRAPPC6B* was identified by Harripaul et al. using microarray and WES in 18 families where there was more than one individual with intellectual disability and parental consanguinity [62]. In this analysis, a nonsense pathogenic variant for *TRAPPC6B* was identified, NM_001079537.2(*TRAPPC6B*):c.124C>T;p.(Arg42Ter). More variants in *TRAPPC6B* have since been reported. Marin-Valencia et al. reported a homozygous splice site pathogenic variant, NM_001079537.2(*TRAPPC6B*):c.150-2A>G, in intron 2 of the *TRAPPC6B* gene after WES of 5000 families with at least one child with a neurological disorder in regions where consanguinity rates were approximately 50% [63]. This variant was identified in six unrelated individuals who presented with early-infantile microcephaly, generalized tonic–clonic seizures, global developmental delay, intellectual disability, and language impairment. Patients also presented with decreased head circumference and short stature, as well as features of muscular pathology, including generalized weakness with normal muscle bulk, ataxic gait, and brisk deep tendon reflexes. This variant is located at a well-conserved splice site in the *TRAPPC6B* gene, causing exon 2 skipping, resulting in a frameshift protein. In ClinVar, there are a number of autosomal recessive variants in *TRAPPC6B* reported as pathogenic for a neurodevelopmental disorder with microcephaly, epilepsy, and brain atrophy. Most variants are predicted to result in no functional protein being produced.

To understand the role of TRAPPC6B in neurodevelopment, Marin-Valencia et al. used a zebrafish model to knock down *TRAPPC6B* using a translation-blocking morpholino (MO) and a splice-blocking MO that masked the splice-donor site of exon 2, mimicking the reported human pathogenic variant [63]. *TRAPPC6B* translation-blocked morphants showed a decreased survival rate, and *TRAPPC6B* splice-blocked morphants had a reduced head size but normal body length, as well as an increased number of apoptotic cells. The seizure threshold and neuronal excitability of *TRAPPC6* knockdown zebrafish were tested under increasing concentrations of pentylenetetrazol (PTZ) to induce seizures. *TRAPPC6* knockdown morphants showed abnormal behavior under all tested conditions and had lower PTZ-induced seizure thresholds and more frequent and intense calcium transients. These results suggest that *TRAPPC6B* pathogenic variants contribute to impaired brain development and neuronal hyperexcitability.

#### 3.1.8. TRAPPC14

TRAPPC14, also known as Microtubule-Associated Protein 11 (MAP11), is a component of TRAPPII. This protein is ubiquitously expressed and plays a role in tethering pre-ciliary vesicles to the mother centriole in ciliogenesis by binding to Rabin8 [64]. Using genome-wide linkage analysis and WES, Perez et al. identified a truncating pathogenic variant in *TRAPPC14* that was associated with autosomal recessive primary microcephaly [65]. In this study, CRSIPR/Cas-9 *TRAPPC14* knockout zebrafish showed microcephaly and decreased neuronal proliferation. The role of TRAPPC14 as a microtubule-associated protein was further elucidated, where immunofluorescence and co-immunoprecipitation studies showed TRAPPC14 association with mitotic spindles and α-tubulin during mitosis.

### 3.2. TRAPPII-Specific Subunits

#### 3.2.1. TRAPPC9

TRAPPC9 was first identified as an NF-kappa-B-inducing kinase (NIK) and IKKbeta binding protein [66], and was consequently named NIBP. In 2007, TRAPPC9 was identified as an essential subunit in the TRAPP family of proteins and is present in almost every sequenced eukaryotic genome [67]. TRAPPC9 is part of the TRAPPII complex, along with TRAPPC10. TRAPPC9 plays a role in NF-kappa B signaling. TRAPPC9 is ubiquitously expressed, but has higher levels in the brain, thyroid, and kidney. NF-kB is a transcription factor that regulates genes involved in inflammation, apoptosis, and synaptic plasticity, and NIK and IKKbeta are signaling molecules involved in two pathways that activate NF-kB (Figure 2). NIK is involved in neurite formation and preventing apoptosis [68], and dysfunction of NIK has been linked to progressive neurological abnormalities and hind-leg paralysis in mice [69]. In the mouse brain and spinal cord, strong TRAPPC9 staining was seen in both the cell body and processes of neurons of the pyramidal layer of the cortex, spinal cord motor neurons, and white matter neurons. Importantly, in the nervous system, *TRAPPC9* is expressed predominantly in neuronal cell types [66]. The importance of TRAPPC9 in neurite elongation was demonstrated by siRNA knockdown of *TRAPPC9* in PC12 neuronal cells, which caused abnormal neuronal differentiation. The role of TRAPPC9 in cell survival was further investigated by measuring *Bcl-xL* gene expression after overexpression and knockdown of *TRAPPC9*. Bcl-xL is an anti-apoptotic gene required for cell survival during development and is regulated by NF-kB signaling. Overall, this study provided detailed insights into the role of TRAPPC9 as an NIK- and IKKbeta-binding protein and its importance in neuronal development and survival.

##### Neurodevelopmental Disorder

Many different pathogenic variants have now been reported in *TRAPPC9* and in autosomal recessive intellectual disability syndrome, also known as NIBP syndrome [70,71,72,73,74,75,76,77,78,79,80,81,82,83]. Patients with NIBP syndrome present with truncal obesity, microcephaly, facial dysmorphism, and autism spectrum disorder. NIBP syndrome is a heterogenous disorder, with numerous variants in *TRAPPC9* identified to date, and has been reviewed previously [7,84]. ClinVar lists over 70 different pathogenic and likely pathogenic autosomal recessive variants that span the gene and cover a wide range of variant types. Variants were associated with intellectual disability, abnormality of the nervous system, brain malformations, autism spectrum disorder, and facial dysmorphism.

##### Cancer

*TRAPPC9*, like *TRAPPC4*, has been shown to be upregulated in multiple cancer types [55,56,57,85,86,87,88,89,90]. In 2021, Cai et al. [91] highlighted TRAPPC9 as a potential drug target for endothelial inflammatory disease due to its role in Nf-kB-mediated inflammation.

#### 3.2.2. TRAPPC10

TRAPPC10 is a subunit in TRAPPII and interacts with the TRAPP core protein, TRAPPC2L [39].

##### Microcephaly

The first report of pathogenic variants in *TRAPPC10* was in patients with microcephaly, short stature, and speech delay who had an autosomal recessive homozygous missense variant NM_003274.5(*TRAPPC10*):c.2786C>T;p.(Pro929Leu), but no functional studies were conducted [92]. In 2022, Rawlins et al. identified compound heterozygous variants in *TRAPPC10* in eight patients from one family with a severe microcephalic neurodevelopmental disorder [93]. They identified a candidate variant, NM_003274.5(*TRAPPC10*):c.3392del;p.(Gly1131fs), resulting in a frameshift variant predicted to cause nonsense-mediated decay. Two siblings in an unrelated family who had a similar phenotype were also reported to carry a homozygous exon 18 variant NM_003274.5(*TRAPPC10*):c.2786C>T;p.(Pro929Leu). ClinVar lists three autosomal recessive nonsense or frameshift variants which are considered likely pathogenic and are associated with a neurodevelopmental disorder with microcephaly, short stature, and speech delay, but no functional evidence was provided to support these.

A yeast two-hybrid assay was used to investigate the effect of the above variants in *TRAPPC10* on interactions with TRAPPC2L, where mutant TRAPPC10 proteins showed reduced TRAPPC2L interaction. A lymphoblastoid line was then generated from one patient with the TRAPPC10 p.(Gly1131fs) variant to study the effect on the TRAPP complexes, where reduced levels of TRAPPC2, TRAPPC2L and TRAPPC3 were observed. Intriguingly, there was an absence of full-length TRAPPC9, indicating a loss of TRAPPII-specific proteins. However, TRAPPIII-specific proteins were unaffected [93]. Furthermore, a VSVG-GFP ts045 trafficking assay performed on HEK293 *TRAPPC10* knockout cells showed a delay in ER-to-Golgi trafficking. Transfection with the wild-type *TRAPPC10* transcript restored the function of this pathway.

A *Trappc10^tm1b(EUCOMM)Wtsi^* mouse model was used to investigate the neuroanatomical features of TRAPPC10-related microcephaly disorder. White matter structures were reduced in size, where size reductions in the anterior commissure and internal capsule were correlated with reduced myelination, but the oligodendrocyte population and density were unaffected [93]. This suggests that in both humans and mice, TRAPPC10 deficiency-related microcephaly is predominantly due to the loss of white matter myelin biogenesis. Since TRAPPC10 patients had short stature, mice were also examined for changes to bone structures. Long bone mass was significantly reduced in females, but overall body length was unaffected.

### 3.3. TRAPPIII-Specific Subunits

#### 3.3.1. TRAPPC8

TRAPPC8 is part of the TRAPPIII complex and is ubiquitously expressed, with high expression in the testis. Recently, a new role for TRAPPIII in ciliogenesis was identified, where TRAPPC8 and TRAPPC12 interact with a specific ciliopathy protein, Oral–Facial–Digital syndrome 1 (OFD1) [94]. TRAPPC8 depletion reduced cilia length and decreased the association of OFD1 with another ciliopathy protein, PCM1.

To date, there are no known disease associations for *TRAPPC8,* and there are no pathogenic or likely pathogenic variants listed in ClinVar.

#### 3.3.2. TRAPPC11

TRAPPC11 is a subunit of the TRAPPIII complex. TRAPPC11 is relatively ubiquitously expressed.

##### Limb Girdle Muscular Dystrophy and Neurodevelopmental Disorder

The first report of a variant in *TRAPPC11* was made in 2013, where Bogershausen et al. linked recessive variants to a disease spectrum of Limb Girdle Muscular Dystrophy (LGMD) and myopathy, as well as intellectual disability [95]. Three individuals from one consanguineous Syrian family presented with LGMD, and five individuals of Hutterite descent presented with myopathy, ataxia, infantile hyperkinetic movements, and intellectual disability. Affected individuals had either a homozygous NM_021942.6(*TRAPPC11*):c.2938G>A;p.(Gly980Arg) missense variant or a homozygous NM_021942.6(*TRAPPC11*):c.1287+5G>A splice site variant, resulting in the loss of exons 11 and 12 and a 58-amino-acid in-frame deletion [95]. Golgi dispersal was seen in fibroblasts from affected individuals, and immunoblot analysis showed a reduction in the full-length TRAPPC11 protein. A yeast two-hybrid analysis was used to demonstrate that the mutant TRAPPC11 protein lost its ability to interact with the TRAPP core, particularly TRAPPC2. To understand the effect of these variants on ER-to-Golgi trafficking, VSV-G-ts045-GFP was used as a marker of this pathway, which showed delayed exit from the Golgi in patient-derived fibroblasts compared to control cells. A significant change in the localization pattern of the late endosomal/lysosomal component lysosome-associated membrane protein 1 (LAMP1) was seen in patient fibroblast cells, and immunoblotting showed a reduction in LAMP1 levels in patient fibroblasts, indicating potential autophagic defects.

Following the seminal *TRAPPC11* variant report, many more *TRAPPC11* variants were identified [96,97,98,99,100,101,102,103,104,105], and in ClinVar, there are over 60 pathogenic or likely pathogenic autosomal recessive variants reported. The reported patients presented with a combination of LGMD, myopathy, intellectual disability, fatty liver disease, and early-onset cataracts [104,105]. Muscle biopsies, including post-mortem biopsies, have shown severe hypoglycosylation in affected individuals, determined via immunoblotting for alpha-dystroglycan [99,103]. However, immunoblots using patient fibroblasts have shown normal labeling of alpha-dystroglycan [99,103], suggesting that the role of TRAPPC11 in ER-to-Golgi alpha-dystroglycan processing is tissue-specific.

In 2021, Irvin et al. completed an exome-wide association study of left ventricular mass, left ventricular diastolic dimension, and relative wall thickness in 1364 participants of African ancestry in the Hypertension Genetic Epidemiology Network and identified rare coding variants contributing to disease, with *TRAPPC11* noted as a candidate [106]. Knockdown of *TRAPPC11* in human-induced pluripotent stem cell (iPSC) cardiomyocytes demonstrated fewer alterations in hypertrophic marker expression and calcium handling compared to controls [106].

Patients with *TRAPPC11* variants have been reported with a variable range of co-morbidities, including myopathy, fatty liver disease, early onset cataracts, neurodevelopmental disorder, microcephaly, seizures, short statures, and intellectual disability, as reviewed recently [107]. Intracellular trafficking defects have been reported for many of the TRAPP subunits, including TRAPPC11. However, distinct from other TRAPP subunits, TRAPPC11 has a role in protein glycosylation. It is required for glycosylation in zebrafish, and deficiency causes an unfolded protein response, lipid accumulation, motility defects, and fatty liver disease [108], as is seen in the human disorder. Depletion of *TRAPPC11* in human cells leads to protein hypoglycosylation [108], and hypoglycosylation specifically of alpha-dystroglycan has been identified in the skeletal muscle and brains of TRAPPC11 patients [103]. Recent evidence has highlighted differential expression of N-glycosylation markers ICAM-1 and LAMP2, markers of congenital disorders of glycosylation in *TRAPPC11*-deficient fibroblasts [107]. Together, this evidence suggests that the unique limb girdle muscular dystrophy phenotype associated with *TRAPPC11* variants may be attributed to the N-linked glycosylation defect, and TRAPPC11 may be considered a congenital disorder of glycosylation.

#### 3.3.3. TRAPPC12

TRAPPC12 is part of the TRAPPIII complex and is ubiquitously expressed, with its highest expression in the testes.

##### Neurodevelopmental Disorder

In 2017, a recessive *TRAPPC12* variant was first linked to childhood encephalopathy, as well as fragmented Golgi in patient fibroblasts and delays in the ER-to-Golgi transport system [109]. Since then, multiple reports have identified other pathogenic *TRAPPC12* variants, all of which cause a spectrum of neurological symptoms including hearing loss, seizures, hypotonia, dystonia, severe global developmental delay, scoliosis, and appendicular spasticity [110,111,112]. Other than the membrane trafficking defects in patient fibroblast studies caused by *TRAPPC12* variants [109], little is known about the pathophysiology of this spectrum of childhood encephalopathies. ClinVar lists 21 pathogenic and likely pathogenic variants, with a range of variant types including frameshift, nonsense, and splicing variants. Most are associated with an early-onset progressive encephalopathy–hearing loss–pons hypoplasia–brain atrophy syndrome or severe hydrocephalous.

#### 3.3.4. TRAPPC13

TRAPPC13 is part of the TRAPPIII complex. TRAPPC13 is relatively ubiquitously expressed, with higher expression in the testis. There are no specific disease associations with the TRAPPC13 subunit, and there are no pathogenic or likely pathogenic variants reported in ClinVar.

## 4. TRAPP Complex, Rab Proteins and Human Genetic Disease

**Figure 3:**
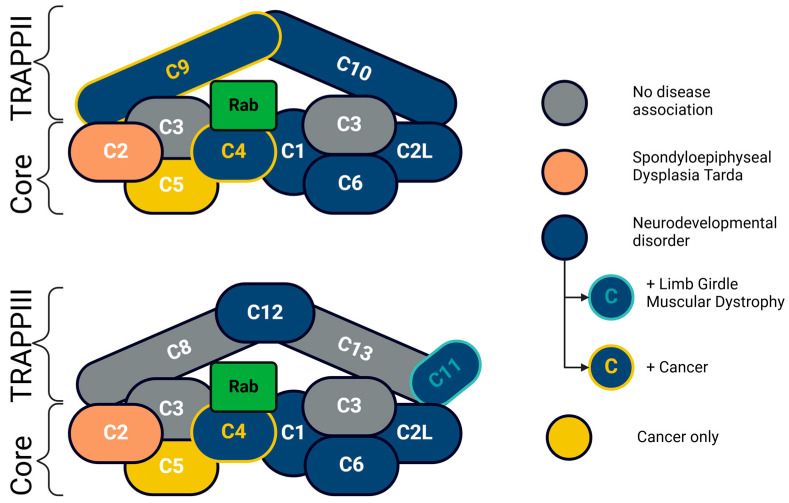
TRAPP complex and disease associations. Pathogenic variants in protein subunits of the core (TRAPPC2L, TRAPPC4, and TRAPPC6) and subunits specific to the two TRAPP complexes, TRAPPII (TRAPPC9 and TRAPPC10) and TRAPPIII (TRAPPC11 and TRAPPC12), are associated with neurodevelopmental disorders. In addition, pathogenic variants in *TRAPPC11* are also involved in Limb Girdle Muscular Dystrophy. Dysregulation of TRAPPC4 and TRAPPC9 also occurs in cancer. The only subunits associated with a clinically distinct phenotype are pathogenic variants in *TRAPPC2*, part of the TRAPP core complex, causing a distinct skeletal disorder, Spondyloepiphyseal Dysplasia Tarda (SEDT). TRAPP complex composition adapted from [114]. Created in BioRender [115].

Despite pathogenic variants in many TRAPP complex protein subunits causing predominantly neurological conditions, except in some cases (Table 1, Supplemental Table S1), phenotypes are protein-specific, meaning they can show varying (but still largely overlapping) phenotypes (i.e., TRAPPC2-skeletal phenotypes, TRAPPC11-muscle phenotypes), and these variants occur across both TRAPPII and TRAPPIII (Figure 3). The underlying mechanisms common to these human phenotypes remain unclear. The location of the variant does not yield any strong association of disease phenotypes with either the TRAPP core, TRAPPII, or TRAPPIII specifically (Table 1, Figure 3); however, neurological disorders are a frequent association with over 50% of the TRAPP complex subunits having reported pathogenic variants. Only TRAPPC2 is associated with a skeletal disorder, whilst TRAPPC11 patients have a multisystem disorder with limb girdle muscular dystrophy and a range of other co-morbidities, including neurodevelopmental disease.

Both TRAPPII and III are GEFs for Rab11 and Rab1, respectively. Rab proteins are a large family of GTPases involved in a diverse range of cell functions from intracellular membrane trafficking to cell signaling, survival, and cell division, and are involved in a diverse range of human diseases [113]. Rab proteins are regulated by their GEFs, for example, the TRAPPII and TRAPPIII complexes, which control both their interaction with membranes and their activation state. When active, they recruit their downstream effectors to the membrane. Specific subunits of TRAPPII and TRAPPIII mediate the Rab substrate specificity, where the extended arms of TRAPPII- or TRAPPIII-specific subunits interact with each RAB and determine Rab activation, as reviewed recently [4]. Both TRAPP protein subunits and Rab proteins are associated with neurodegenerative diseases. Neurons, with their extended morphology and long life, have unique requirements for efficient intracellular trafficking and regulation of autophagy.

Generally, Rab11 and TRAPPII regulate the late secretory pathway of Golgi-to-plasma membrane trafficking via secretory vesicles. In synapses, Rab11 is a modulator of synaptic transmission [116], and it is likely that this may be mediated by TRAPPII regulation of neuronal function and maintenance of the synaptic cycle [117]. These processes are essential for neuronal health. Rab11 also regulates the insertion and removal of neurotransmitter receptors at the plasma membrane in neurons [118]. Pathogenic variants in *Rab11* are associated with the neurodegenerative disease Alzheimer’s disease and affect the regulation of β-amyloid production [119]. Rab11 also interacts with α-synuclein and prevents its aggregation, a process that is involved in the progression of Parkinson’s disease (PD) [120]. Interestingly, aggregated TRAPPC6A can also cause Alzheimer’s disease by triggering caspase activation, Tau aggregation, and β-amyloid generation, leading to a cascade of protein aggregation linked to neurodegeneration [121].

Rab1 is expressed in all tissues, is present in all eukaryotes, and is considered a housekeeping protein. However, it is the interaction of Rab1 with effectors or other proteins that affects Rab1 function and localization. Activation of Rab1 is mediated by its GEF, the TRAPPIII complex, specifically at the TRAPPC8 subunit. Rab1 and TRAPPIII, regulate trafficking between the ER and Golgi and the formation of autophagosome membranes during autophagy. Rab1 is directly linked to the pathogenesis of PD and other neurodegenerative diseases [122] as a substrate of leucine-rich repeat kinase 2 (LRRK2), in which variants are the most common cause of familial and sporadic PD [123].

Rab18 is another Rab GTPase activated by the TRAPPII complex [124], and is recruited to lipid droplets, Golgi, ER, endosomes and peroxisomes [125,126]. Although the exact role of Rab18 in the cell is largely unknown, recent studies suggest that it plays a role in organelle tethering, autophagy, and the mediation of lipid droplet dynamics [127,128,129]. Upon TRAPPII depletion, the recruitment of Rab18, but not Rab1, to lipid droplets was defective [124]. TRAPPII interacts with COPI, which regulates lipid droplet homeostasis, and this interaction facilitates Rab18 activation. Impairment of TRAPPII-mediated Rab18 results in abnormally large lipid droplets and defective lipogenesis [124]. Rab18 deficiencies lead to Warburg–Micro syndrome [130], which is characterised by neurodevelopmental delay, microcephaly, and congenital cataracts [131].

## 5. Conclusions and the Future of New Disease Models

Most TRAPP subunits are highly conserved from yeast to humans and are essential for viability (Table 1). There are a number of conserved functions between TRAPP subunits such as vesicular transport from the ER to the Golgi, GEF exchange, and autophagy (Supplemental Table S1). However, some components, including TRAPPC11 and TRAPPC13, are not present in yeast, indicating that these TRAPP components may have unique functions in humans aside from their core role in ER-to-Golgi trafficking, secretion, and autophagy, and may have tissue specificity (Figure 1; Supplemental Table S1). For example, the TRAPPC2 subunit is the only subunit where pathogenic variants lead to a skeletal disorder, whilst TRAPPC11 has a unique limb girdle muscular dystrophy phenotype in humans. It is therefore likely that TRAPP complex subunits, like many proteins, function in multiple roles and cause distinct clinical phenotypes.

Recent advances in genetic diagnostic technologies have made it possible to identify TRAPP variants and link them to phenotypes seen in the clinic. While accurate diagnosis is a critical aspect of good clinical care, further steps must be made to understand the pathophysiological processes behind TRAPPopathies in order to enable the development of effective therapeutics and to further our understanding of some of the unique cellular functions of certain subunits. Many TRAPP variants have been associated with neurological and muscular pathologies, but there are very few disease models that have been generated to study TRAPP disorders. Currently, most functional genomic modeling of TRAPP complex pathogenic variants has been undertaken in patient-derived cells, primarily skin fibroblasts. Overexpression/knockdown studies have been conducted in human cell models such as HEK293T cells, or in yeast homologues of the human proteins. These models can provide important insights at a cellular level of perturbation, including in trafficking and autophagy, and are useful tools to confirm the pathogenicity of novel variants. However, they do not mimic the affected cell type, for example, neurons or skeletal muscle cells.

The rapid expansion of new technologies in gene editing, high-resolution sequencing techniques including single cell RNA sequencing, stem cell-based disease modeling, directed differentiation, and organoid culture, will enable the development and analysis of in vitro models that contain the relevant tissue types to study TRAPPopathies and related disorders. This will allow us to recapitulate the disease genetics in iPSC either derived from patients or engineered to carry gene mutations, and in the future may open avenues for exploring new targeted therapies. For example, to study neurological dysfunction, directed differentiation approaches by incorporation of specific proneural transcription factors into iPSC allow for the rapid generation of relatively homogenous populations of neuronal cell types [132,133]. These models, which can be derived from patient iPSCs, can then be used to study disease mechanisms and for drug screening [134]. However, these two-dimensional monolayer cultures lack the architectural complexity and cell diversity found in vivo. In contrast, brain organoid models, three-dimensional aggregates derived from pluripotent stem cells, are able to self-organize and develop some of the cellular diversity (which include distinct neural progenitor, neuronal, and glial cell types) and architectural organization of the developing human brain [135]. Brain organoids have been used to study human brain development and the neurobiology underlying complex human neurodevelopmental diseases [136,137,138]. Additionally, iPSC can be differentiated into chondrocytes to provide a relevant substrate for the study of the defective chondrogenesis in TRAPPC2-related disorders [139]. Lastly, skeletal muscle cells can be derived from iPSC to study limb girdle muscular dystrophies [140] for the study of TRAPPC11-related disorders. In all, iPSCs provide a renewable source of cells to generate a wide range of cell types in order to study a range of genetic conditions. They have significant value in our understanding of underlying disease mechanisms and in the development of potential therapies for affected individuals.

## Figures and Tables

**Table 1:** Overview of disease associations with the TRAPP complex.

Human Subunit	Yeast Homologue	TRAPP Complex Component	Essential for Yeast Viability	OMIM Disease Association *	Inheritance Pattern
C1	Bet5p	core	Y	Neurodevelopmental delay, hypotonia, lower limb spasticity (but not listed in OMIM)	AR
C2	Trs20p	core	Y	Spondyloepiphyseal Dysplasia Tarda (SEDT)-(MIM 313400)	XLR
C2L	Tca17p	core	Y	Encephalopathy, progressive, early-onset, with episodic rhabdomyolysis (MIM 618331)	AR
C3	Bet3p	core	Y	No reported disease associations	NA
C4	Trs23p	core	Y	Neurodevelopmental disorder with epilepsy, spasticity, and brain atrophy (MIM 618741); cancer association	AR
C5	Trs31p	core	Y	Hepatocellular carcinoma	NA
C6	Trs33p	core	N	Neurodevelopmental disorder with microcephaly, epilepsy, and brain atrophy (MIM 617862)	AR
C9	Trs120p	TRAPPII	Y	Intellectual developmental disorder, autosomal recessive 13 (MIM 613192); cancer association	AR
C10	Trs130p	TRAPPII	Y	Neurodevelopmental disorder with microcephaly, short stature, and speech delay (MIM 620027)	AR
C14/MAP11		TRAPPII		Microcephaly 25, primary, autosomal recessive (MIM 618351)	AR
C8	Trs85p	TRAPPIII	N	No reported disease associations	NA
C11	Not present in yeast	TRAPPIII		Muscular dystrophy, limb-girdle, autosomal recessive 18 (MIM 615356)	AR
C12	Not present in yeast	TRAPPIII		Encephalopathy, progressive, early-onset, with brain atrophy and spasticity (MIM 617669)	AR
C13	Trs65p	TRAPPIII	N	No reported disease associations	NA

OMIM, Online Mendelian Inheritance in Man; MIM, Mendelian Inheritance in Man phenotype number; AR, autosomal recessive; XLR, X-linked recessive; NA, not applicable. * MIM database accessed November 2024.

## Supplementary Materials

The supporting information can be downloaded at: https://www.mdpi.com/article/10.3390/ijms252413329/s1.
